# Primary malignant melanoma, an atypical presentation in the cervical spine: a case report

**DOI:** 10.1186/s13256-023-04290-5

**Published:** 2023-12-17

**Authors:** Larrey Kasereka Kamabu, Louange Maha Kataka, Bives Mutume Nzanzu Vivalya, Franck Katembo Sikakulya, Hervé Monka Lekuya, Moses Galukande

**Affiliations:** 1 Department of Surgery, Neurosurgery, College of Medicine, Makerere University, Kampala, Uganda; 2 Faculty of Medicine, Université Catholique du Graben, Butembo, Democratic Republic of the Congo; 3 Department of Internal Medicine, Masereka General Hospital, Goma, North-Kivu Democratic Republic of the Congo; 4 Department of Psychiatry and Mental Health, Kampala International University Western Campus, Ishaka, Uganda; 5 Department of Surgery, Kampala International University, Western Campus, Ishaka, Uganda; 6 Directorate of Surgical Services, Neurosurgical Unit, Mulago National Referral Hospital, Kampala, Uganda

**Keywords:** Malignant melanoma, Cervical spine, MRI

## Abstract

**Background:**

Few studies have documented the occurrence of melanoma in the cervical spine. Of all malignant melanoma cases, 1% are primary melanoma of the central nervous system, which makes it extremely uncommon and nonspecific. We aim to report a case of the uncommon presentation of primary melanoma in the cervical spine.

**Case presentation:**

The patient was a 59-year-old Muganda male who presented with a 2-year history of anterior neck swelling as well as severe pain and a tingling sensation in the left shoulder and arm, which worsened in the recent 6 months. He developed weakness and paresthesia in the upper left arm and progressive gait disturbance of the left leg. A physical examination revealed masses in the left cervical and right submandibular region. Additionally, the upper and lower left extremities revealed hemiparesis and hemihypoesthesia. A magnetic resonance imaging scan showed a hyperintense lesion on TIWI and another hypointense lesion on T2WI, originating from the cervical spine and involving the vertebral bodies and paravertebral soft tissues. The patient underwent surgery, a black tumor was extracted, and histology revealed the tumor to be malignant melanoma. The patient died within 1 month after the diagnosis and surgery.

**Conclusion:**

This case is presented to highlight the significance and challenges associated with making a pre- and postoperative diagnosis of primary cervical melanoma with atypical radiological characteristics. Patients with extradural lesions that show hyperintensity on T1-weighted images and hypointensity on T2-weighted images should have spinal melanoma examined as a possible differential diagnosis.

## Introduction

Primary malignant melanoma in the cervical spine is an exceptionally rare and distinct entity worth investigating [1]. Primary malignant melanoma, which originates from the central nervous system (CNS), represents roughly 1% of the total melanoma cases [2]. Unlike other spinal tumors, its occurrence is infrequent, making it challenging to diagnose and treat [3, 4]. In this case report, we aim to provide a concise and focused overview of this unique condition, shedding light on its rarity, distinctive features, and the potential implications for clinical management.

Reporting unusual presentations of primary malignant melanoma in the cervical spine is of paramount importance for several reasons. First, the rarity of this condition means that there is limited existing knowledge and literature on its clinical characteristics and management [3]. Each reported case adds valuable information to the medical community and contributes to a better understanding of the disease [2].

Second, the challenges associated with both the preoperative and postoperative diagnosis of the disease make it crucial to document and share such cases [5]. Unusual radiological features in the primary malignant melanoma of the cervical spine can mimic other more common spinal tumors, leading to misdiagnosis or delayed diagnosis [5]. When determining the presence of spinal tumors, magnetic resonance imaging (MRI) is the preferred technique. This method can be used to show how spinal malignant melanoma presents itself. Fat deposits are hyperintense on T1-weighted images (T1WI) as well as on T2-weighted images (T2WI), and the presence of melanin as well as acute or chronic intratumoral hemorrhages affects the MRI signaling of melanocytic tumors [4]. The most effective management of malignant melanoma is a wide excision. The use of chemotherapy and radiotherapy is the subject of ongoing debate, and their effects may vary from patient to patient [4]. Accurate preoperative diagnosis is essential for appropriate surgical planning and decision making. [5]

Furthermore, the postoperative diagnosis and histopathological evaluation of primary malignant melanoma remain complex due to the rarity and unfamiliarity of its presentation [6]. Clinicians and pathologists may not always be adequately prepared to handle such cases, and therefore, reporting them helps to raise awareness and to enhance diagnostic accuracy in the future [6].

In conclusion, this case report aims to provide a focused overview of primary malignant melanoma in the cervical spine, emphasizing its rarity and unique characteristics. By documenting this unusual presentation and addressing the challenges associated with preoperative and postoperative diagnosis based on atypical radiological features, we seek to contribute valuable insights to the medical literature. Through a better understanding of this condition, our report may have a significant impact on improving the diagnosis and management of primary cervical melanoma, ultimately leading to enhanced patient care and outcomes.

## Case presentation

A 59-year-old Muganda male presented to the Neurosurgery Department of the Mulago National Referral Hospital with a 2-year history of anterior neck swelling, severe pain, and tingling sensations in the left shoulder and arm. His symptoms worsened in the recent 6 months; he developed weakness and paresthesia in the left upper arm and experienced progressive gait disturbance of the left lower limb. He denied any familial history of cancer.

## Clinical findings

The patient was afebrile and did not exhibit pallor. On examination, a soft mass measuring approximately 6 cm × 5 cm was found in the left anterior neck and appeared to be attached to the underlying structures. Additionally, another mass was noted in the right submandibular region, measuring 3.8 cm × 2.5 cm. This mass was rubbery in texture and was not attached to the underlying tissues, indicating an enlarged submandibular lymph node.

Neurological examinations revealed hemiparesis of the left leg and arm, hemihypoesthesia with hyperreflexia and a positive Babinski sign in the left leg, impaired sensation of position, vibrations in the left side, and impaired cerebellar tests.

## Timeline

The patient had noticed his anterior neck swelling over 2 years, associated with severe pain and a tingling sensation in the left shoulder and arm.

As his symptoms worsened, he consulted the Neurosurgery Department of Mulago National Referral Hospital 6 months later, when he developed weakness and paresthesia in the upper left arm and progressive gait disturbance in the left leg.

## Diagnostic assessment

The patient underwent preoperative laboratory investigations, which included complete a blood count and blood chemistry, and the findings were within normal limits. Serology for human immunodeficiency virus (HIV) was also negative.

## Radiological imaging

**Fig. 1:**
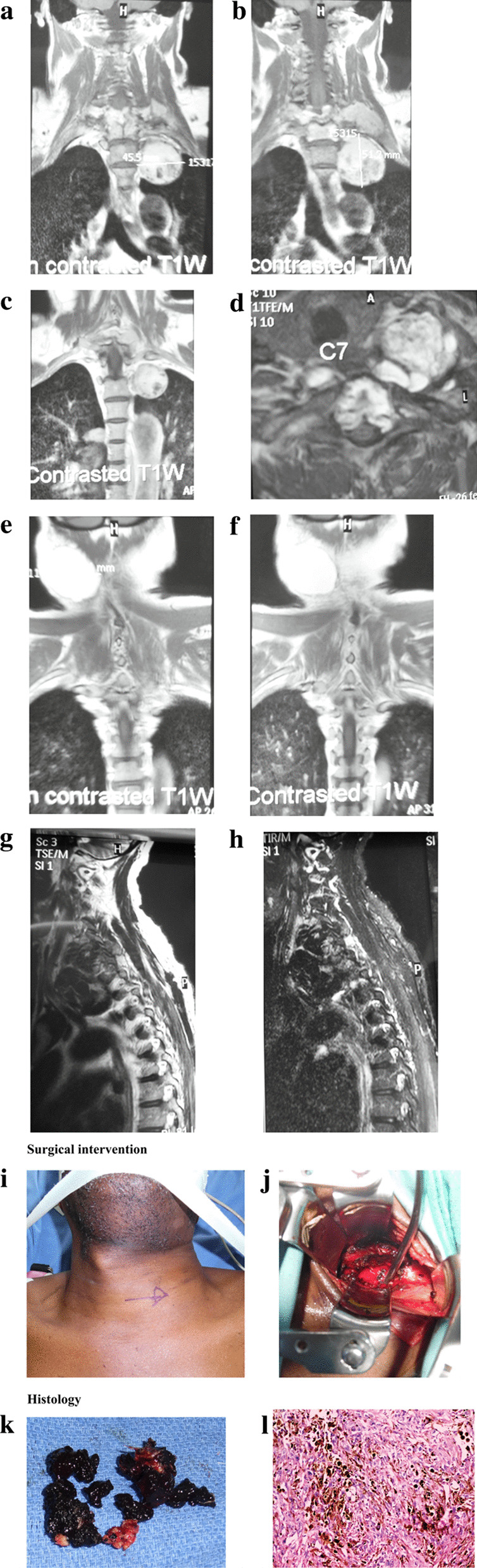
**a** Noncontrasted coronal TIWI MRI showing a well defined hyperintense lesion in the thoracocervical region measuring 5.12 cm × 4.55 cm. **b** Noncontrasted coronal TIWI MRI showing measurement of the lesion. **c** Contrasted coronal TIWI MRI showing no contrast enhancement of the lesion. **d** Axial TIWI showing the same lesion with infiltration of the surrounding tissues and the disc with associated narrowing of the spinal canal. The trachea is displaced to the right. **e** Noncontrasted coronal TIWI showing an enlarged submandibular lymph node. **f** Contrasted coronal TIWI showing no contrast enhancement of the submandibular node. **g** Sagittal T2WI showing a well-defined hypointense lesion in the thoracocervical region (C5-T1). **h** A sagittal STIR image hypointense lesion in the thoracocervical region. **i** A picture showing the region of the mass preoperatively (arrow). The vocal cord is displaced to the right. **j** An intraoperative picture showing the site of the tumor and residual tumor (black). **k** Picture showing black tumor extracted during surgery. **l** Histology hematoxylin and eosin (H&E) stain × 100 M showing the tumor composed of spindle to polygonal cells having abundant intra- and extracellular pigment

The MRI scan showed a disruption in the cervical curve and alignment. There was a well-defined extradural lesion, hyperintense on TIWI and hypointense on T2WI, and short tau inversion recovery (STIR). The lesion showed no enhancement on the contrast MRI scan (using a gadolinium based contrast agent) and extended from the C5 to T2 vertebral levels. The image revealed infiltration into the surrounding tissues and the vertebral body heights of C5–T1 were reduced with irregular margins. The bone marrow signal intensities were increased in TIWI for the same vertebrae. The C7/T1 discs had reduced signal intensities on T2WI and protruded into the spinal canal, which was narrowed due to compression from the cord at that level. The paravertebral soft tissue and the trachea were displaced to the right. A solitary hyperintense (TIWI) nonenhancing mass was seen in the right submandibular region measuring 3.1 cm × 2.5 cm (sentinel lymph node; Fig. 1a–h).

Radiological diagnosis of a neoplastic process was made with differential diagnosis of lymphoma, metastases, and vascular malformation. The chest X-ray and abdominal ultrasound scans were normal.

## Discussion

### Therapeutic intervention

#### Surgical intervention

After being brought to the operating room, the patient underwent general anesthesia. Baseline motor and sensory potentials evoked motor responses in the left leg. On the operating table, the patient was lying supine with his head secured in a Mayfield head holder. A sterile dressing and preparation of the anterior cervical area were made. An incision was made transversely, and the dura was cut and stitched back with Nurolon sutures. After that, microscissors were used to cut the underlying arachnoid matter. Bipolar cautery was used to seal off the midline raphe, and an 11-blade scalpel was used to cut the pia mater. The aberrant tissue was reached in a few millimeters using a midline dissection with microinstruments. The operation allowed for a total excision of a black mass, in total measuring 3.1 cm × 2.5 cm from the extradural region of the C5–T1 level (Fig. 1i, j). Several bridging vessels had to be cauterized and cut with a pair of microscissors before the tumor could be removed from the spinal cord. Around the perimeter of the resection cavity, the vertebral bodies and discs were infiltrated and appeared black. An anterior osteotomy was done. The Medtronic centerpiece set was then used to implant the C5–T1 laminoplasty. At each level, plates and screws were employed to attach the lamina to the lateral mass. A running 4–0 Nurolon suture was used to seal off the dura. Following that, interrupted 0 and 2–0 Vicryl sutures were used to close the deep and surface fascial layers, and the skin was stapled shut. The excised mass was taken for histology.

An anterior approach was used for the removal of the anterior cervical spine primary melanoma. This approach involves accessing the cervical spine from the front of the neck, allowing direct access to the tumor and surrounding structures.

The technique employed for the surgical resection of the tumor, using an anterior approach, typically includes the following steps:

Incision: a small horizontal or oblique incision is made in the front of the neck to access the cervical spine.

Dissection: the surgeon carefully separates the muscles and tissues to expose the anterior aspect of the cervical spine, vertebral bodies, and intervertebral discs.

Exposure: after exposing the affected area, the tumor is visualized along with its relationship to nearby critical structures, such as the spinal cord, nerve roots, and major blood vessels.

Tumor resection: the surgeon carefully excises the malignant melanoma along with a margin of healthy tissue to ensure complete removal and to reduce the risk of recurrence.

Reconstruction: depending on the extent of the tumor resection, spinal stability may be compromised. In this case, the Medtronic centerpiece set can be used to implant the C5–T1 laminoplasty. At each level, plates and screws should be employed to attach the lamina to the lateral mass to restore stability and maintain proper alignment of the spine.

Closure: after the tumor has been resected and any necessary spinal reconstructions have been performed, close the incision and suture the wound.

#### Histology

Multiple black, coarse, and friable tissue bites were resected and sent to histology. The histological examination revealed spinal cord tissue with a tumor that was composed of proliferating polygonal to spindle shaped cells with an abundance of intracytoplasmic brown-to-black pigment. Vesicular nuclei with noticeable nucleoli were present in the tumor cells, which were positioned in tightly packed sheets and clusters. There were numerous mitotic figures and unusual mitosis of the cells. Focused observations revealed strange, large, multinucleated cells, and there were also areas of necrosis and bleeding. The Fontana Masson (FM) stain revealed the presence of a melanin pigment. More specifically, the intracytoplasmic pigment was melanin, as determined by melanin bleach. The histopathology of the right submandibular mass revealed that the lymph node had been infiltrated by cancer (Fig. 1k, l).

The identification of the intracytoplasmic pigment as melanin was a crucial step in confirming the diagnosis of primary malignant melanoma in the cervical spine. Histological staining techniques, particularly the FM stain, play a significant role in this identification process.

The FM stain is a special histochemical staining method used to demonstrate the presence of melanin pigment in tissue sections. The staining is done in the following steps:

Tissue preparation: tissue samples obtained from the tumor resection are fixed and embedded in paraffin blocks to create thin sections suitable for microscopic examination.

Deparaffinization: the paraffin-embedded tissue sections are deparaffinized by immersing them in xylene, followed by rehydration through a graded series of alcohol.

Incubation: the tissue sections are then subjected to a series of reagents, including silver nitrate and hydroquinone (Fontana’s silver reduction method), which reacts with melanin to produce a visible stain.

Reduction of silver: the silver ions in the staining solution are reduced to metallic silver by the melanin granules present in the tissue. This reduction reaction results in the deposition of black or brownish-black silver granules, specifically within the melanin-containing cells.

Counterstaining: to provide contrast and improve tissue visualization, the sections are counterstained with a suitable dye, such as nuclear fast red.

Under the microscope, the melanin granules will appear as darkly stained, coarse, or fine particles within the cytoplasm of melanocytes or melanoma cells. The presence of these characteristic pigmented granules confirms the diagnosis of melanin-producing tumor cells.

The use of the FM stain is particularly valuable in distinguishing primary malignant melanoma from other tumors in the cervical spine, which may have overlapping histological features but lack melanin pigmentation. By identifying the intracytoplasmic melanin pigment, the staining technique provided definitive evidence of the tumor’s melanocytic origin, contributing to the accurate diagnosis of primary malignant melanoma in the cervical spine.

### Follow-up and outcomes

On the third postoperative day, the patient was extubated, as the tumor was not completely removed. After the tumor resection, the patient’s strength and sensibility gradually returned to Medical Research Council (MRC) grade 4/5; however, the patient eventually developed a neurogenic bladder, so an in-and-out catheterization was needed. After achieving mean arterial pressure objectives of > 85 mmHg on day 10 for 10 days, the patient was moved out of the intensive care unit, and, on postoperative day 25, he was sent to an acute rehabilitation facility.

In the postoperative follow-up, the patient was thoroughly examined by ophthalmology, dermatology, and gastrointestinal specialists to investigate the possibility of a primary cause of melanoma, but no significant findings were observed. He also had follow-up appointments with radiation oncology and hematology–oncology to plan for adjuvant radiation therapy and immunotherapy using nivolumab and ipilimumab, respectively, after his condition was stabilized. Unfortunately, the patient desaturated and aspirated prior to discharge, as his condition destabilized. Despite cardiopulmonary resuscitation, the patient passed away a month after surgery. Patients diagnosed with metastatic cutaneous melanomas normally have a 1-year survival rate, but initial spinal cord melanomas have a 6-year and 7-month survival rate [7, 8]. However, our patient had been complaining of anterior cervical spine swelling for 2 years. We believe that if the patient had been consulted earlier and received timely treatment, the tumor extraction may have been lifesaving and overall promote improved quality of life and survival.

## Discussion

This case describes an uncommon presentation of primary melanoma in the cervical spine. In addition, it highlights the importance and challenges of preoperative diagnosis of primary cervical melanoma with unusual radiological features. Furthermore, the case reveals a comprehensive discussion of the patient’s clinical course and treatment response, factors that may have contributed to the poor outcome, and available treatment options, as well as other limitations and supporting evidence and studies.

### Clinical features and diagnosis

Primary melanoma is rare condition, specifically in the cervical spinal cord, with scant information available for its clinical and radiological diagnosis, treatment, and prognosis [9]. Melanomas that most frequently develop in the skin, mucous membranes, leptomeninges, brain parenchyma, and uvea contain melanocytes [4]. The intramedullary, leptomeningeal, and dural origins of the primary malignant melanoma were made clear. When primary CNS melanoma has a spinal location, it typically manifests in the thoracic portions. [10]. The clinical picture is vague and points to a gradual cord compression. In this case study, the primary melanoma developed from the cervical spine. There have been less than 70 documented cases of melanoma, specifically in the cervical spinal cord, worldwide, highlighting its rarity [9, 11]. We have not performed immunohistochemistry on the tissue due to limited resources and the following reasons, which represent the realities of spinal tumor diagnosis and care in Sub-Saharan Africa: (1) the patient could not afford it, (2) postoperative staging was not done, and (3) routine molecular testing is not available in Uganda. This case report has some limitations, such as we have been unable to incorporate the BRAF status of the primary cervical melanoma in this case report due to unavailability of molecular testing and epigenetic profiling of malignant melanoma in Uganda. We recognize that incorporating the BRAF status could provide valuable information to other clinicians and researchers, enabling them to understand the genetic characteristics of this rare presentation and contributing to the advancement of personalized medicine for melanoma patients.

### Imaging modalities

When determining the presence of spinal tumors, MRI is the preferred technique, as it allows for accurate imaging of internal structures such as spinal malignant melanoma. Melanin, acute or chronic intratumoral hemorrhages, and fat deposits all affect the MRI signal of melanocytic tumors [12]. The existence of persistent organic radicals inside melanin allows it to exhibit paramagnetic activity in the typical melanotic melanoma. The unpaired electrons of these free radicals interact with water protons, shortening the T1 and T2 relaxation durations, further resulting in hyperintensity on T1-weighted images and on T2-weighted images. This patient’s MRI results showed hyperintensity on TIWI and hypointensity on T2WI, which is consistent with previous research and denotes a high melanin concentration [11].

In T1-weighted images, amelanotic melanoma and melanoma without a hemorrhagic component show isointense–hyperintense and moderately hyperintense, respectively. Preoperatively, it might be challenging to identify melanoma from other tumors such as meningioma, lipoma, and metastases. The most frequent consequence of a metastatic lesion is damage in the spine [13]. Although this was the situation in this patient, metastasis was less expected because the patient had no original tumor.

Other imaging techniques have a limited relevance in the imaging of primary spinal malignant melanoma. A simple radiograph can indicate bone damage and help locate the disease. Although computed tomography does not show bone marrow edema, it does indicate bone involvement better than MRI.

### Prognosis

According to previous research, primary spinal melanoma progresses more slowly and is less aggressive than the more common melanoma of the skin that has evidence of metastasizing in the central nervous system [4]. The average survival duration of patients with primary spinal malign melanoma is 6–7 years. [14] In this instance, although the patient underwent surgery, as shown in Fig. 1i–k, the patient did not recover and passed away a month following the diagnosis.

Given the low incidence and wide range of possible manifestations of primary spinal melanoma, there is no established treatment for this disease. Many authors concur that the optimum course of action is, whenever practical, full surgical excision. The use and effectiveness of chemotherapy and radiotherapy use are still in debate [4].

### Available treatment options and their benefits and limitations

There has not been much investigation into the treatment and prognosis of primary spinal cord melanoma. Resection has been proposed as the preferred treatment for primary spinal melanomas [7, 15]. Subtotal resection was the second most popular method used for resection after gross total resection (GTR) [15]. Although the survival period is yet uncertain, there have been theories that whole-body excision and postoperative radiation might contribute to its extension. Leptomeningeal seeding and hydrocephalus are unfavorable prognostic indicators that indicate disease development and make it harder to achieve complete melanoma excision [16].

Radiation therapy with a dose of 12 to 40 Gy may also be an option [9]. In approximately 78% of cases, metastatic disease was not detected, even though 40% of the patients had received chemotherapy or radiation therapy [8, 9]. Radiation is the usual course of treatment for CNS melanoma after surgical resection [21, 22]. Initial spinal cord lesions have a better prognosis than metastatic melanomas, despite the fact that metastatic melanoma in the CNS develops rapidly and is resistant to adjuvant therapy. In total, 20% of the instances reported by Khan *et al*. in the chosen studies resulted in death, while the majority of the treated patients returned to their regular lives after therapy [15]. Furthermore, immunotherapy has been proposed in treating primary spine melanoma, with limed data documenting its use in this pathology [9]. Different chemotherapeutic agents including tenozolomide, dacarbazine and levamisol, procarbazin, CCNU, and vincristin have been used in management of primary spine melanoma with varying results [17–20]. Depending on the location, size, severity of the symptoms, and the patient’s tolerance, gross total resection may be curative. Due to the small number of cases, the range of treatments used, and the varying follow-up duration, we are unable to comment on the efficacy of different therapies for the treatment of recurrence and metastasis.

Targeted therapy: for melanomas with specific genetic mutations, targeted therapies such as vemurafenib and dabrafenib combined with trametinib have shown promising results. These drugs specifically target mutated proteins in melanoma cells, inhibiting their growth and proliferation.

Combination therapies: some cases may benefit from a combination of different treatment modalities, such as immunotherapy with targeted therapy or radiation therapy in specific situations.

In summary, wide excision remains the primary and most effective management approach for malignant melanoma. However, the role of chemotherapy and radiotherapy in the treatment of melanoma is a subject of ongoing debate and may vary based on individual cases. The management of malignant melanoma is highly individualized, considering factors such as the stage of the disease, tumor characteristics, genetic mutations, and the overall health of the patient. Research in this field is ongoing, leading to the development of novel therapies and treatment strategies that continue to improve patient outcomes.

## Conclusion

This case report set out to describe the unusual presentation of primary malignant melanoma and MRI features of primary malignant melanoma. It is presented to highlight the importance and challenges of pre- and postoperative diagnosis of primary cervical melanoma with unusual radiological features. In patients with extradural lesions with hyperintensity on T1-weighted images and hypointensity on T2-weighted images, spinal melanoma should be considered as a differential diagnosis. A viable therapeutic strategy includes local treatment combined with surgical resection and radiation, according to the body of existing evidence.

## Data Availability

Not applicable.
